# Multi-drug resistant Acinetobacter species: a seven-year experience from a tertiary care center in Lebanon

**DOI:** 10.1186/s13756-017-0297-6

**Published:** 2018-01-22

**Authors:** Zeina A. Kanafani, Nada Zahreddine, Ralph Tayyar, Jad Sfeir, George F. Araj, Ghassan M. Matar, Souha S. Kanj

**Affiliations:** 1 Department of Internal Medicine, Division of Infectious Diseases, American University of Beirut, Cairo Street PO Box 11-0236/11D, Riad El Solh, Beirut, 1107 2020 Lebanon; 2 Infection Control and Prevention Program, American University of Beirut, Cairo Street PO Box 11-0236/11D, Riad El Solh, Beirut, 1107 2020 Lebanon; 3 Department of Pathology and Laboratory Medicine, American University of Beirut, Cairo Street PO Box 11-0236/11D, Riad El Solh, Beirut, 1107 2020 Lebanon; 4 Department of Experimental Pathology, Microbiology, and Immunology, American University of Beirut, Cairo Street PO Box 11-0236/11D, Riad El Solh, Beirut, 1107 2020 Lebanon

**Keywords:** Acinetobacter, Intensive care unit, Ventilator-associated pneumonia, Multi-drug resistance, Lebanon

## Abstract

**Background:**

*Acinetobacter* species have become increasingly common in the intensive care units (ICU) over the past two decades, causing serious infections. At the American University of Beirut Medical Center, the incidence of multi-drug resistant *Acinetobacter baumannii* (MDR-Ab) infections in the ICU increased sharply in 2007 by around 120%, and these infections have continued to cause a serious problem to this day.

**Methods:**

We conducted a seven-year prospective cohort study between 2007 and 2014 in the ICU. Early in the epidemic, a case-control study was performed that included MDR-Ab cases diagnosed between 2007 and 2008 and uninfected controls admitted to the ICU during the same time.

**Results:**

The total number of patients with MDR-Ab infections diagnosed between 2007 and 2014 was 128. There were also 99 patients with MDR-Ab colonization without evidence of active infection between 2011 and 2014. The incidence of MDR-Ab transmission was 315.4 cases/1000 ICU patient-days. The majority of infections were considered hospital-acquired (84%) and most consisted of respiratory infections (53.1%). The mortality rate of patients with MDR-Ab ranged from 52% to 66%.

**Conclusion:**

MDR-Ab infections mostly consisted of ventilator-associated pneumonia and were associated with a very high mortality rate. Infection control measures should be reinforced to control the transmission of these organisms in the ICU.

## Background

Multidrug-resistant organisms (MDRO) have significant infection control implications and are currently affecting the clinical course of patients in tertiary care centers. *Acinetobacter baumannii* is of particular importance. The organism is widely distributed in nature and survives on moist and dry surfaces [1, 2]. Worldwide, multidrug-resistant *A. baumannii* (MDR-Ab) has become a significant cause of hospital-acquired infections (HAI) and hospital-acquired colonizations (HAC) resulting in high morbidity and mortality [3] in patients admitted to the intensive care units (ICU) over the past two decades [4]. Strict adherence to infection control practices and environmental disinfection have been effective in controlling outbreaks [5]. Appropriate strategies and practices must therefore be implemented to prevent the growing transmission of MDR-Ab.

In line with the worldwide emergence of MDR-Ab, similar trends have been observed at various centers in Lebanon. Although national studies are lacking, the available evidence suggests rapidly falling susceptibility rates to carbapenems (from 49.2% in 2011 to 15.1% in 2013 at 16 selected hospitals) [6], and a predominance of OXA-23 and GES-11 with upstream insertion sequence ISAba1 (90% of isolates in a study from 11 centers) [7]. At the American University of Beirut Medical Center (AUBMC), HAI and HAC caused by MDR-Ab initially increased in the ICU in 2007 from 2-3 cases to 5-6 per month. These infections were mostly associated with invasive devices such as ventilators, central venous catheters, and urinary catheters. Investigations carried by the Infection Control and Prevention Program (ICPP) identified multiple factors that contributed to the transmission of MDR-Ab. We herein describe our experience at the AUBMC with MDR-Ab over a 7-year period and the infection control measures that were implemented to control the spread of this organism in the ICU.

## Methods

### General description and settings

AUBMC is a 386-bed teaching tertiary care center functioning as a referral center at the national and regional levels. The ICU consists of a medical and surgical unit with a nine-bed capacity. Three single- and three double-bed rooms are spread around a central nursing station. The ICU population consists of high-risk patients with multiple comorbidities, as well as patients following major surgical procedures. The beds in the double rooms are maintained at a distance of 3 m and separated by textile curtains.

### Study design

A 7-year prospective cohort study was conducted in the ICU with systematic attempts to assess present practices and to introduce new interventions to contain the transmissions of MDR-Ab in the unit. All ICU patients were evaluated examining the risk factors attributed to the transmission of MDR-Ab HAI or HAC. The ICU team routinely collected specimens from newly admitted patients for baseline bacteriological studies and all patients were placed under contact isolation. A standardized screening method was adopted, where cultures were obtained from deep tracheal aspirates (DTA), urine, oropharyngeal, axillary, umbilical, perianal, and rectal areas. The ICPP team reviewed the culture results on daily basis to advise on the isolation status of patients through daily surveillance rounds. Patients identified with MDR-Ab were kept on contact isolation. Cultures were repeated on weekly basis until discharge. All MDR-Ab HAI and HAC were periodically discussed with the ICU staff for feedback and interventions. Furthermore, environmental cultures were obtained following the identification of clusters or outbreaks from the direct environment of the patient and from the medical equipment used inside the ICU cubicle. Repeated cleaning and disinfection was performed for all surfaces or equipment identified to be contaminated with MDR-Ab. A nested retrospective case-control study from January 2007 till June 2008 was performed in the ICU and the Respiratory Care Unit (RCU) to analyze patient related risk factors leading to MDR-Ab transmissions. Controls were randomly selected from patients admitted to the ICU and the RCU during the same study period but who did not have a positive screening culture for MDR-Ab. Moreover, cases consisted of patients with one or more cultures growing MDR-Ab (either colonized or infected). For patients with multiple MDR-Ab culture results, only the first positive culture was considered. The data were entered into a database using IBM® SPSS® Statistics version 21.

### Definitions

According to the Centers for Disease Control and Prevention (CDC), a multidrug-resistant pathogen is defined as one that is resistant to one or more classes of antimicrobial agents, including carbapenems [5]. In this study, MDR-Ab was defined as an isolate that is resistant to all tested antimicrobial agents except colistin and tigecycline [8]. A culture positive for MDR-Ab was considered to represent colonization when patients showed no evidence of infection. As for the case definition, patients with at least one clinical/surveillance specimen positive for MDR-Ab were defined as cases of transmission of MDR-Ab colonization or infection that was not present on admission. Such patients were considered to have acquired MDR-Ab during their ICU stay. For device-associated infections, the definitions were subject to considerable variation since 2005 based on the updates issued by the CDC and when the reports published by the National Nosocomial Infections Surveillance (NNIS) system were updated and replaced by the National Healthcare Safety Network (NHSN). All infections were classified using the CDC definitions of the corresponding year using laboratory and clinical criteria. Infection control staff collected data on central line-associated primary bloodstream infections (CLABSI), ventilator-associated pneumonias (VAP), and urinary catheter associated urinary tract infections (CAUTI) in patients admitted to the adult ICU. Corresponding ICU denominator data consisting of patient-days and device-days were also collected by infection control staff for the same calendar month [9, 10].

### Description of clusters and outbreaks

A cluster was defined as an aggregation of MDR-Ab cases (more than 2 cases), closely grouped in time and place. When the number of cases in the cluster exceeded 4 transmissions, it was considered an outbreak.

### Organism identification and susceptibility testing

*Acinetobacter* isolates were identified using the MALDI-TOF platform for identification. All isolates were tested using the disk diffusion method based on the Clinical and Laboratory Standards Institute (CLSI) breakpoints. The colistin sensitivity testing was made based on VITEC-2 Bio System and disk diffusion according to the method reported by Galani et al. in 2008 study [11, 12].

### Ethical considerations

A written informed consent was not needed for our study as the information was obtained from the daily surveillance rounds of the ICPP team. The medical records of patients were routinely reviewed as part of the ongoing ICPP work. Patients were not contacted and medical records were not retrieved a second time to write the manuscript. Over the years, statistics were collected and stored for statistical analysis and periodic reports within the institution. All figures included in the manuscript were retrieved from the preexisting ICPP files without having to perform a review of patient records. Furthermore, the available statistics did not include any identifiable information to maintain patient confidentiality.

## Results

### Results of the case-control study of Acinetobacter infections 2007–2008

A total of 73 cases infected with *Acinetobacter spp*. (carbapenem-sensitive and resistant Acinetobacter isolates) and 73 controls (uninfected patients) were included. The mean age of the infected patients was 61.7 ± 17.7 years with a male predominance (male:female ratio of 2:1). Culture specimens consist mostly of respiratory secretions (58%), followed by wound (22%), blood (12%), and urine (8%). Moreover, the microbiological distribution of the isolates was predominated by one species, namely *Acinetobacter baumannii* complex (70 isolates, 96%) with the other 4% distributed between *A. junii* (2 isolates, 2.7%) and *A. lwoffi* (1 isolate, 1.3%). In addition, 40 patients (55%) had carbapenem-resistant isolates, 26 of which were tested against colistin and found to be susceptible. Most infections were deemed to be hospital-acquired (84%) with only 16% being community-acquired. Furthermore, underlying comorbidities such as diabetes mellitus, renal insufficiency, chronic obstructive pulmonary disease (COPD), and malignancy were significant risk factors for developing an Acinetobacter infection. In addition, patients who had undergone surgical interventions and those who received antibiotics within 30 days prior to admission were at significant risk for developing an Acinetobacter infection (*p* < 0.05) (Table 1).

**Table 1:** Bivariable analysis of patient characteristics in the case-control study

Variable	Cases (n = 73)	Controls (*n* = 73)	Unadjusted Odds Ratio	*p*-value
Age (mean ± SD), *in years*	61.7	60.4	N/A	0.798
Male	49 (67.1)	52 (71.2)	0.82	0.591
Diabetes	42 (57.5)	10 (13.7)	8.53	< 0.001
Chronic pulmonary disease	35 (47.9)	12 (16.4)	4.68	< 0.001
Renal insufficiency	32 (43.8)	12 (16.4)	3.97	< 0.001
Malignancy	21 (28.8)	7 (9.6)	3.81	0.005
Corticosteroid intake	5 (6.8)	7 (9.6)	0.69	0.55
Urinary catheter in the past 30 days	62 (84.9)	58 (79.4)	1.46	0.39
Central venous catheter in the past 30 days	7 (9.6)	4 (5.5)	1.83	0.35
Mechanical ventilation in the past 30 days	43 (58.9)	39 (53.4)	1.25	0.50
Surgery in the past 30 days	19 (26.0)	5 (6.8)	4.78	0.003
Antibiotic use in the past 30 days	47 (64.3)	12 (16.4)	9.19	< 0.001
All-cause mortality	34 (46.6)	27 (37.0)	1.48	0.24

All numbers represent no. (%) unless otherwise specified

SD = standard deviation; N/A = not applicable

All complications due to Acinetobacter infections, except for acute respiratory distress syndrome (ARDS), were encountered more with resistant strains as compared to sensitive ones, but none of these complications was of statistical significance (Table 2).

**Table 2:** Complications and outcome in patients with susceptible *Acinetobacter* infection vs. MDR-Ab infection in the case-control study

Variable	Susceptible *Acinetobacter* infection (*n* = 33) n (%)	MDR-Ab infection (*n* = 40) n (%)
Sepsis	15 (45.4)	17 (42.5)
ARDS	2 (6.1)	0
Respiratory failure	4 (12.1)	8 (20.0)
ICU admission	5 (15.1)	8 (20.0)
AKI	8 (24.2)	10 (25.0)
Prolonged hospital stay	27 (81.2)	31 (77.5)
Persistence/progression of infection	5 (15.1)	13 (32.5)
Recurrence of infection	6 (18.2	8 (20.0)
All-cause mortality	12 (36.4)	22 (55.0)

MDR-Ab = multidrug-resistant Acinetobacter; ARDS = adult respiratory distress syndrome; ICU = intensive care unit; AKI = acute kidney injury

### Results of the prospective study of MDR-Ab transmissions in ICU 2007–2014

The total number of patients with Acinetobacter infections diagnosed between 2007 and 2014 was 128 (Table 3). There were also 99 patients with MDR-Ab colonization without evidence of active infection between 2011 and 2014. Prior to 2011, screening of patients on admission to the ICU was not performed.

**Table 3:** Characteristics of the patients infected with MDR-Ab in the prospective study of MDR-Ab transmissions in ICU 2007–2014

Clinical Characteristics		Number of patients	Percent
Gender	Male	77	60.2
	Female	51	39.8
Age	> 70 years	54	42.2
	≤ 70 years	74	57.8
Diabetes		42	32.8
Chronic pulmonary disease		86	67.2
Hemodialysis		21	16.4
Malignancy		38	29.7
Past surgical procedures		46	36.0
Recent mechanical ventilation		73	57.0
In-ICU mortality		28	22.0
Carbapenem susceptibility	Susceptible	33	25.8
	Resistant	95	74.2

MDR-Ab = multidrug-resistant Acinetobacter; ICU = intensive care unit

The mean age of the 128 patients was 58.3 years (range 19–96) with a male predominance (60.2%). The mean length of ICU stay was 3.6 days (range 1–14 days). Outliers for patients staying for more than 30 days (maximum recorded length of stay was 5 months) were documented but were not included in the calculation of the mean length of stay (3 patients). The most common site of infection among the isolates was the respiratory tract (53.1%), followed by surgical wound (18.8%), blood (15.6%), urine (10.2%) and others (2.3%) (Table 4). The most common colonization site among the 99 cases was the respiratory tract (80.8%) followed by skin colonization (12.4%). The mortality rate (22%) in the ICU was associated with old age, trauma, cancer, multiple comorbidities, and invasive device use.

**Table 4:** Types of MDR-Ab infections in ICU between 2007 and 2014

Year	CLABSI	VAP	SSI	CAUTI	Others	Total
2007	2	11	0	0	3	16
2008	3	14	3	3	0	23
2009	2	6	3	3	0	14
2010	1	8	8	1	0	18
2011	3	7	5	0	0	15
2012	3	11	3	2	0	19
2013	4	5	1	0	0	10
2014	2	6	1	4	0	13
Total number (%)	20 (15.6)	68 (53.1)	24 (18.8)	13 (10.2)	3 (2.3)	128

MDR-Ab = multidrug-resistant Acinetobacter; ICU = intensive care unit; CLABSI = central line associated bloodstream infection; VAP = ventilator-associated pneumonia; SSI = surgical site infection; CAUTI = catheter-associated urinary tract infection

During the outbreak period from December 2012 to December 2014, 130 patients out of 1267 (10.3%) admitted to the ICU became colonized or infected with MDR-Ab, with patients from the surgical ICU having slightly less risk than those from the medical ICU. The overall colonization pressure (number of MDR-Ab patient-days × 1000/total number of patient-days) of MDR-Ab between 2012 and 2014 was 315.4 cases per 1000 ICU patient-days (range 262.8–361.6) (Table 5). In addition, the average length of stay for MDR-Ab patients admitted to the ICU was 9.7 days (range 1–150) with the average length of stay till the acquisition of MDR-Ab being 7.3 days (range 2–31). The all-cause mortality rate of patients dying with MDR-Ab infection/colonization was high and ranged between 52% and 66%. Given the fact that the patients were critically ill, calculating the attributable mortality was challenging.

**Table 5:** Colonization pressure among patients in ICU during 2012–2014

Year	2012	2013	2014
MDR-Ab days	1130	814	925
Patient days	3125	3097	2873
Colonization pressure per 1000 patient days	361.6	262.8	322.0

ICU = intensive care unit; MDR-Ab = multidrug-resistant Acinetobacter

Moreover, the ICPP took several control measures to help break the transmission cycle of the organism. Hand hygiene, universal screening and isolating all newly admitted patients played a key role in containing the outbreaks. Furthermore, the change in cleaning protocols and the extensive focus on educating healthcare workers limited the spread of MDR-Ab to other hospital wards. Didecyldimethylammonium chloride (DDAC) was adopted for cleaning and disinfection of floors, walls, surfaces, and medical devices. This disinfectant and detergent has bactericidal and fungicidal activity, in addition to being active on HCV, HIV-1, and influenza virus at a dilution of 0.25% (20 ml in 8 l of water).

Table 6 summarizes the clusters and outbreaks encountered throughout the study period along with control measures that were undertaken by the ICPP:

**Table 6:** General characteristics of the reported clusters and outbreaks in ICU

Timeline	Characteristics			Identified source	Control measures	Recurrence
1995–2007	Scattered clusters affecting 1–2 patients/month			• Endogenous • Common source	• Hand hygiene compliance • Patient placement on contact isolation	Intermittent
2007–08	Outbreaks occurring in ICU on periodic basis Incidence rate 5–25/1000 patient-days			Case-control study • Endogenous - Malignancy - Recent surgery • Common source - Water contamination with MDR-Ab	• Targeted intervention - Infection control practices - Enforcing adherence to hand hygiene - Proper use of personal protective equipment - Controlled visitation to patients - Targeted education and guidance • Intensified presence of ICPP team	Frequent
2009–11	Major outbreaks occurring in ICU on monthly basis Incidence rate 4–30/1000 patient-days			• Endogenous - Underlying diseases - Invasive procedures • Common source - Contaminated ventilators Lack of compliance with IC measures	Key measures • Strict compliance hand hygiene policy • Patient placement on contact precautions until cleared by negative results of screening cultures • Judicious use of antibiotics • H_2_O_2_ decontamination	Ongoing
2012–14	Alternating endemic clusters and outbreaks occurring on monthly basis Colonization pressure:			Significant findings for point and propagated sources	Implementation of Drastic measures were implemented - Key revisions of policies and procedures - Key change in cleaning and disinfection methods	Ongoing
	2012	2013	2014			
	284.0	263.0	322.0			

### Environmental cultures

Sampling environmental culture swabs from patients’ environment and equipment was conducted throughout the study period. As a result, positive cultures were recovered from samples taken from the ventilators, the portable X-Ray and the nitric oxide machines. By molecular typing, these isolates were found to be identical to the bacteria isolated from the patients. These pieces of equipment were thought to play a major role in the outbreak. Subsequently, ICPP proposed new protocols for the process of placing patients on assisted respiratory therapy and issued detailed procedures for cleaning and disinfection of ventilators. Cultures were taken from additional environmental sources including the water, the faucets, and the air conditioning outlets in the rooms and failed to yield any Acinetobacter growth. Other sources that were identified during the investigation of later outbreaks included leaking mattresses and pillows, which were thought to be also possible reservoirs for MDR-Ab. All leaking mattresses and pillows were discarded and replaced by new ones.

### Colonization pressure

The program adopted tracking the MDR-Ab colonization pressure (CP) and reporting it on a monthly basis. In fact, during the same study period, MDR-Ab CP was relatively high and correlated with the high crude numbers of MDR-Ab infections and colonizations. By that time, transmissions of MDR-Ab had become endemic.

### Bundles approach

Additional steps that became standard of care in the nursing units included the implementation of the bundles for device-associated infections (VAP, CLABSI, and CAUTI bundles) as recommended by the Institute for Health Care Improvement, proper monthly training for healthcare workers especially in the critical care units, adoption of a “bare below elbow” outfit for all ICU workers, and daily presence of the ICPP team members in the ICUs. All these measures were essential to containing the spread of MDR-Ab inside the ICU. The addition of close-circuit television (CCTV) cameras was also instrumental in identifying health care personnel breaches during the evening and night shifts. These cameras had an additive effect and contributed to the control of the epidemic.

## Discussion

Over the past decade, *Acinetobacter spp*. have been increasingly associated with hospital infections and colonizations. Our study describes several outbreaks caused by MDR-Ab between 2007 and 2014. Our initial case control study of Acinetobacter infections, between 2007 and 2008, revealed that most of the infected patients were elderly, with a male predominance, similar to the study by Abbo et al. [13]. Positive cultures consisted mostly of respiratory secretions, followed by wound, blood, and then urine; findings comparable to an international study [14]. *Acinetobacter baumannii* was the predominant isolated species with only few isolates of *A. junii* and *A. lwoffi*. At the beginning of the study about half of the isolates were carbapenem resistant, of which around half were found to be susceptible to colistin. Most of the infections were considered hospital-acquired with a small percentage being community-acquired infections.

As in previously reported studies [15], patients infected with Acinetobacter had several risk factors including underlying comorbidities such as diabetes mellitus, renal insufficiency, COPD, and malignancy. Furthermore, surgical interventions and prior antibiotic treatment within 30 days before admission were also found to be significant risk factors for developing an Acinetobacter infection in concordance with a study by Playford et al. [16].

In this study, we compared infections with susceptible versus resistant isolates and found that there was a trend towards more sepsis, respiratory failure, ICU admission, and prolonged hospital stay in infections with MDR-Ab strains. However, acute respiratory distress syndrome (ARDS) was seen in both groups. Similar findings were seen in another study in the ICU from China [17].

In the prospective study conducted between 2007 and 2014, there was also a predominance of male gender, with a mean age of 60 years comparable to our case-control study. The mean length of stay in the ICU was around 4 days, however, outliers for patients staying for more than 30 days were documented. During this period, carbapenem resistance among Acinetobacter isolates increased steadily, with prevailing MDR-Ab towards the end of 2014. This was likely due to the significant increase in carbapenem use at AUBMC, in view of the rising incidence of extended spectrum Beta lactamase producing Enterobacteriacae [18]. The most common site of infection among the patients with Acinetobacter infections was found to be the respiratory tract, followed by surgical wound, blood, and urine as reported in other studies [19]. Similarly, the most common colonization site between 2011 and 2014 was the respiratory tract followed by skin colonization.

Acinetobacter infections have been associated with increased mortality in several published reports. In our study, the all-cause mortality rate of patients with MDR-Ab infection/colonization was high, but it was difficult to calculate the attributable mortality due to the fact that many patients were critically ill with multiple comorbid conditions. Higher mortality rates were seen in older patients, those with trauma, cancer, multiple comorbidities, and invasive device use.

In addition, during the study period, the average length of stay for MDR-Ab patients admitted to the ICU increased. Patients with Acinetobacter incurred greater financial costs than those who did not have Acinetobacter transmissions. It is estimated that a single ventilator-associated pneumonia (VAP) or central line-associated bloodstream infection (CLABSI) due to MDR-Ab may result in 2 weeks of additional hospitalization with its incurred added cost. The average cost of ICU stay, at our medical center, for one patient with MDR-Ab infection can reach $1750 per day. For an extended ICU stay of 2 weeks, the patient’s bill can be up to $24,000. Because of the poor outcome of the Acinetobacter infections and the incurred increased morbidity, hospital stay and cost of infected patients, the ICPP adopted a series of control measures since December 2012.

For example, in view of published supportive evidence [20], the use of the H_2_O_2_ vaporizer for room disinfection after discharges of colonized or infected patients was initiated in 2013. Although in this report, the Acinetobacter contamination in the ICU environment was found to be a cause of recurrent MDR-Ab clusters or outbreaks, lack of proper hand hygiene and lack of adherence to proper infection control practices were thought to play a major role in the spread of this organism. Audits conducted by the ICPP team as well as anonymous audits led to the identification of several breaches by the health care providers that were promptly addressed. The nursing director and the chief of staff office issued warnings for health care workers with repeated acts of non-compliance. Finally, changes in the reporting of data, namely the introduction of the CP measure as an important predictor of infection and colonization [21], helped in standardization and benchmarking of infection rates.

Our study has limitations. Patient-level antibiotic treatment data were not available. Therefore, patient outcome could not be analyzed based on treatment received. Colonized patients were not followed after discharge from the ICU. The only outcome available for these patients was the overall mortality rate. Attributable mortality was not assessed because of multiple confounding variables such as underlying illnesses, invasive procedures, cancer patients, etc. Another limitation is that some of the data were obtained retrospectively and could not be re-verified. Finally, the fact that multiple interventions were implemented at the same time in an effort to control the epidemic prevented the analysis of the effect of each measure by itself.

## Conclusion

In conclusion, at our center, MDR-Ab infections mostly caused ventilator-associated pneumonia and were associated with a very high mortality rate. Acinetobacter can colonize several environmental sources including respirators, mattresses and others. It is an organism that is difficult to eradicate and easy to spread in the ICU setting. Adherence to proper infection control measures is key in controlling the transmission and spread of these organisms in the ICU.

## References

[1] Espinal P, Marti S, Vila J (2012). Effect of biofilm formation on the survival of Acinetobacter Baumannii on dry surfaces. J Hosp Infect.

[2] Jawad A, Heritage J, Snelling AM, Gascoyne-Binzi DM, Hawkey PM (1996). Influence of relative humidity and suspending menstrua on survival of Acinetobacter spp. on dry surfaces. J Clin Microbiol.

[3] Sunenshine RH, Wright MO, Maragakis LL (2007). Multidrug-resistant Acinetobacter infection mortality rate and length of hospitalization. Emerg Infect Dis.

[4] Caldeira VM, Silva Junior JM, Oliveira AM (1992). Criteria for patient admission to an intensive care unit and related mortality rates. Rev Assoc Med Bras.

[5] Siegel JD, Rhinehart E, Jackson M, Chiarello L. Healthcare Infection Control Practices Advisory C. Management of multidrug-resistant organisms in health care settings, 2006. Am J Infect Control. 35(10 Suppl 2)):S165–93. (2007) 10.1016/j.ajic.2007.10.006 18068814

[6] Chamoun K, Farah M, Araj G (2016). Surveillance of antimicrobial resistance in Lebanese hospitals: retrospective nationwide compiled data. Int J Infect Dis.

[7] Hammoudi Halat D, Moubareck CA, Sarkis DK (2017). Heterogeneity of Carbapenem resistance mechanisms among gram-negative pathogens in Lebanon: results of the first cross-sectional countrywide study. Microb Drug Resist.

[8] Magiorakos AP, Srinivasan A, Carey RB (2012). Multidrug-resistant, extensively drug-resistant and pandrug-resistant bacteria: an international expert proposal for interim standard definitions for acquired resistance. Clin Microbiol Infect.

[9] Dudeck MA, Edwards JR, Allen-Bridson K (2015). National Healthcare Safety Network report, data summary for 2013, device-associated module. Am J Infect Control.

[10] Edwards JR, Peterson KD, Andrus ML (2007). National healthcare safety network (NHSN) report, data summary for 2006, issued June 2007. Am J Infect Control.

[11] Galani I, Kontopidou F, Souli M (2008). Colistin susceptibility testing by Etest and disk diffusion methods. Int J Antimicrob Agents.

[12] Nhung PH, Miyoshi-Akiyama T, Phuong DM (2015). Evaluation of the Etest method for detecting colistin susceptibility of multidrug-resistant gram-negative isolates in Vietnam. J Infect Chemother.

[13] Abbo A, Navon-Venezia S, Hammer-Muntz O, Krichali T, Siegman-Igra Y, Carmeli Y (2005). Multidrug-resistant Acinetobacter baumannii. Emerg Infect Dis.

[14] Maslow JN, Glaze T, Adams P, Lataillade M (2005). Concurrent outbreak of multidrug-resistant and susceptible subclones of Acinetobacter Baumannii affecting different wards of a single hospital. Infect Control Hosp Epidemiol.

[15] Dizbay M, Tunccan OG, Sezer BE, Hizel K (2010). Nosocomial imipenem-resistant Acinetobacter Baumannii infections: epidemiology and risk factors. Scand J Infect Dis.

[16] Playford EG, Craig JC, Iredell JR (2007). Carbapenem-resistant Acinetobacter Baumannii in intensive care unit patients: risk factors for acquisition, infection and their consequences. J Hosp Infect.

[17] Ye JJ, Huang CT, Shie SS (2010). Multidrug resistant Acinetobacter Baumannii: risk factors for appearance of imipenem resistant strains on patients formerly with susceptible strains. PLoS One.

[18] Araj GF, Avedissian AZ, Ayyash NS (2012). A reflection on bacterial resistance to antimicrobial agents at a major tertiary care center in Lebanon over a decade. J Med Liban.

[19] Bergogne-Berezin E, Towner KJ (1996). Acinetobacter spp. as nosocomial pathogens: microbiological, clinical, and epidemiological features. Clin Microbiol Rev.

[20] Blazejewski C, Wallet F, Rouze A (2015). Efficiency of hydrogen peroxide in improving disinfection of ICU rooms. Crit Care.

[21] Castelo Branco Fortaleza CM (2013). Moreira de Freitas F, da Paz Lauterbach G. Colonization pressure and risk factors for acquisition of imipenem-resistant Acinetobacter Baumannii in a medical surgical intensive care unit in Brazil. Am J Infect Control.

